# A time and motion study of patients presenting at the accident and emergency department at Mater Dei Hospital

**DOI:** 10.1186/1756-0500-4-421

**Published:** 2011-10-18

**Authors:** Matthias Azzopardi, Marija Cauchi, Karl Cutajar, Robert Ellul, Charles Mallia-Azzopardi, Victor Grech

**Affiliations:** 1 Mater Dei Hospital, Msida, MSD2090, Malta; 2 Infectious Diseases Unit, Mater Dei Hospital, Msida, MSD2090, Malta; 3 Paediatric Department, Mater Dei Hospital, Msida, MSD2090, Malta

## Abstract

**Background:**

To carry out a time and motion study of patients presenting at the Emergency Department (ED) by measuring waiting times at the ED dept throughout the day. The objectives were:

• to determine whether waiting times are prolonged, and

• if prolonged, at which station(s) bottlenecks occur most often in terms of duration and frequency.

Results will be compared to the United Kingdom guidelines of stay at the emergency department.

**Methods:**

A group of 11 medical students monitored all patients who attended ED between 0600 hours on the 25^th ^August and 0600 hours on the 1st September 2008. For each 24 hour period, students were assigned to the triage room and the 3 priority areas where they monitored all patient-related activity, movement and waiting times so that length of stay (LOS) could be recorded. The key data recorded included patient characteristics, waiting times at various ED process stages, tests performed, specialist consultations and follow up until admitted, discharged, or referred to another hospital area. Average waiting times were calculated for each priority area. Bottle-necks and major limiting factors were identified. Results were compared against the United Kingdom benchmarks - i.e. 1 hour until first assessment, and 4 hours before admitting/discharge.

**Results:**

1779 patients presented to the ED in the week monitored. As expected, patients in the lesser priority areas (i.e. 2 & 3) waited longer before being assessed by staff. Patients requiring laboratory and imaging investigations had a prolonged length of stay, which varied depending on specific tests ordered. Specialty consultation was associated with longer waiting times. A major bottleneck identified was waiting times for inpatient admission.

**Conclusions:**

In conclusion, it was found that 30.3% of priority 1 patients, 86.3% of priority 2 patients and 76.8% of priority 3 patients waited more than 1 hour for first assessment. We conclude by proposing several changes that may expedite throughput.

## Background

Malta is the largest island of an archipelago situated in the centre of the Mediterranean and the total population approaches half a million. There is one acute general teaching hospital (Mater Dei Hospital - MDH) offering an extensive range of hospital and specialist services at no cost, based on a National Health Service system. This is the only centre in Malta providing critical care services. Health Centres (also on the National Health Service) are also available all around the Island, and these provide non-critical, elective as well as acute care, at no cost. 111, 688 persons attended the Emergency Department in 2007. Prolonged waiting times at the MDH Emergency Department (ED) have been heavily criticised by the public in the media [1]. Naturally, prolonged waiting times lead to public dissatisfaction [2] with the service being offered, and patients are known to leave without waiting to be seen. Moreover, it has been shown that timely care leads to improved patient health outcomes [3]. For these reasons, guidelines of acceptable waiting times at EDs have been developed in the US [4], the UK [5,6], and several other countries. The MDH ED includes one triage room, 3 care areas with 18 treatment cubicles (11 in area 1, 5 in area 2, 2 in area 3), one plaster room, 3 resuscitation areas, X-ray and waiting areas, and a paediatric casualty area.

The staff complement at any one time is around 6 casualty officers, comprised of a mixture of house officers, 1-3 senior registrars/registrars, 1-3 senior house officers and 2-3 consultants. In addition, 2-3 GP trainees may also be present.

Upon arrival and registration at MDH, patients are assessed in the triage room and categorised into three triage categories: life threatening/immediate (priority one), urgent/semi-urgent (priority two), and non-urgent patients (priority three). The latter are patients that could have been seen by a family doctor or at a Health Centre outside the hospital premises. Paediatric medical cases are referred to the paediatric casualty area, whereas patients suffering from gynaecological, ophthalmic and ear, nose and throat problems may be referred to their respective wards if specialist doctors are available to see them.

This study was conducted at the MDH ED, and its aim was to carry out a time and motion study of patients presenting at ED by measuring various staged waiting times. The triage room and the three areas were constantly monitored, 24 hours a day, for a period of one week. Factors contributing to patient care delay were identified, and the effect of independent variables estimated. An attempt was made to identify how ED management can improve to significantly decrease length of stay and thus improve ED patient care efficiency.

## Methods

Patients presenting between the 25/08/08 at 0600 and 01/09/08 at 0600 were monitored from their time of registration at the ED to the time of admittance to a ward, discharge or referral. 1779 patients attended the ED over this time period. Only priority one, two and three patients were considered in this study; patients who were referred to another department were excluded from the study once they left the ED. Children referred to the emergency paediatric department were excluded from the study after leaving triage. A pilot study had been previously conducted on Friday 22/08/08 from 1000 to 1800 to finalise the methodology of data collection. 11 medical students conducted the study round the clock, with 5-6 students being present at any one time. One student was stationed in the triage room, one in area 3, one in area 2 and 2-3 in area one. Three students were present at area one during peak hours (1200-2400). Neither ED staff nor patients were involved in the process of data collection. Ethical approval was obtained from the University of Malta Research and Ethics Committee. Owing to the nature of the audit and the fact that there was no direct contact with patients, the Ethics Committee deemed it unnecessary to obtain written informed consent from the patients.

Two time data sheets were used for each patient entering the ED, with one being filled in the triage room and the other in the respective area. The time of registration of the patient at the ED was noted together with all patient movements and related casualty officer or nurse movements. The following time intervals were recorded for each patient: waiting time to enter triage, triage time, waiting time to enter area, waiting time from registration to first assessment, time taken to be seen by nurses 1-3, waiting time to be seen by casualty officers 1-5, wait for specialist review (defined as the time between referral and being seen by a senior), total time taken for imaging, ECG time, wait to leave ED after admission or discharge, total time in area and ED. Interaction times between patients and carers were calculated from the data gathered. The criteria used for the waiting times depended on steps where patients had to wait for a procedure to be carried out or for staff to see them [7-10]. Other patient characteristics noted were age, sex, nationality, locality, presenting complaint and referral source (health centre, GP, self-referral, Gozo general hospital, police referral). Investigations taken in triage or area, as well as referral to another department were also noted.

Patients with missing data were included where relevant, e.g. if the patient left at any stage before being discharged, his/her waiting times were included until the moment of leaving.

### Statistics

All data collected was split up by time of arrival into four time groups: 0200-0800, 0800-1400, 1400 2000, 2000-0200. The data was also divided into two time groups: between 0200 and 1400, and between 1400 to 0200 hours. This point was selected because it was noted that after 2 pm there were few or no senior registrars present at the emergency department and patients often had to wait for the senior registrar to be called down from the wards before being admitted. Data analysis was performed using Microsoft Excel 2007. Time groups were compared using unpaired T-tests. Quartiles and interquartile ranges (IQR) were calculated using a bespoke Excel spreadsheet. Due to the non-normal distribution of certain data, both means and medians were calculated and presented. A *p *value ≤ 0.05 was taken to represent a statistically significant result.

## Results

During the study period the data of 1779 patients was collected. Of the 1779 patients, 23% (403) were referred to another department from triage. 11% of patients left without being seen after triage, and 2% failed to answer when called for triage. Priorities were assigned thus: 21% to priority 1, 21% to priority 2, and 19% to priority 3. 16% of patients attending the ED were admitted to the wards. 175 patients arrived to MDH by ambulance. 68 of these were non-acute cases.

The median waiting time for all patients was 92 minutes (mean = 115 minutes) and the median length of stay from registration to admittance/discharge was 208 minutes (mean 244 minutes - tables 1, 2 and 3).

**Table 1:** Waiting and interaction times in hours and minutes in priority 1

	NUMBER				MEAN				MEDIAN				IQR				RANGE			
	
	T_1 _	T_2 _	T_3 _	T_4 _	T_1 _	T_2 _	T_3 _	T_4 _	T_1 _	T_2 _	T_3 _	T_4 _	T_1_	T_2_	T_3_	T_4_	T_1 _	T_2 _	T_3 _	T_4 _
Wait To Enter Triage	31	66	68	58	0:07	0:07	0:07	0:12	0:03	0:05	0:05	0:09	0:10	0:10	0:08	0:13	00:00-00:44	00:00-00:40	00:00-00:24	00:00-01:06

Triage Time Taken	33	70	73	61	0:03	0:04	0:04	0:04	0:03	0:04	0:03	0:04	0:03	0:05	0:04	0:03	00:00-00:10	00:00-00:17	00:00-00:19	00:00-00:12

Wait To Enter Area	26	49	56	48	0:27	0:13	0:19	0:25	0:03	0:03	0:02	0:03	0:27	0:06	0:08	0:42	00:00-02:40	00:00-02:29	00:00-04:24	00:00-02:08

Wait From Registration To First Assessment	31	91	83	63	0:53	0:33	0:55	1:17	0:41	0:21	0:36	1:01	1:09	0:25	0:54	1:44	00:00-02:35	00:00-02:45	00:00-03:26	00:04-04:28

Wait To Be Seen By Nurse 1	34	74	59	38	0:45	0:30	0:23	0:47	0:27	0:04	0:06	0:17	0:58	0:59	0:27	0:30	00:00-03:53	00:00-04:52	00:00-03:30	00:00-04:47

Total Interaction Time Nurse	29	71	53	32	0:42	0:38	1:08	1:18	0:22	0:18	0:15	0:28	1:22	0:56	0:55	1:27	00:01-03:23	00:01-03:06	00:01-07:15	00:01-13:20

Wait To Be Seen By CO 1	45	77	110	74	0:26	0:16	0:41	0:50	0:09	0:10	0:19	0:22	0:46	0:29	0:24	0:55	00:00-02:47	00:00-01:37	00:00-03:30	00:00-00:40

Total Interaction Time CO	45	121	103	68	0:21	0:26	0:23	0:21	0:17	0:21	0:18	0:17	0:24	0:22	0:22	0:21	00:03-00:59	00:01-01:46	00:01-01:55	00:01-01:08

Time First Seen To Time Last Seen By CO	45	121	106	67	1:19	1:28	1:17	1:16	0:57	1:10	0:48	0:28	1:38	1:15	1:38	1:29	00:01-10:20	00:01-08:56	00:01-08:15	00:01-10:33

Wait For Senior Review	12	34	22	9	0:49	1:05	1:32	1:34	0:40	0:47	1:26	0:39	1:34	0:26	1:09	1:23	00:05-01:46	00:00-03:38	00:00-04:57	00:14-04:57

Total Time X-ray Taken	26	95	78	44	0:14	0:18	0:20	0:16	0:13	0:13	0:17	0:11	0:14	0:15	0:19	0:09	00:03-00:44	00:03-01:28	00:04-01:05	00:04-01:20

Total Time CT Taken	1	9	6	7	0:15	0:18	0:18	0:15	0:15	0:14	0:10	0:18	-	0:17	0:15	0:21	00:03-01:28	00:05-00:42	00:06-01:02	00:08-00:26

Total Time US Taken	2	5	11	3	0:11	0:19	0:27	0:08	0:11	0:21	0:16	0:08	-	0:22	0:19	-	00:09-00:13	00:14-00:26	00:07-01:17	00:08-00:09

Total Treatment Time	11	19	20	15	0:45	0:46	0:55	0:31	0:14	0:18	0:30	0:09	0:41	1:04	1:01	0:47	00:02-02:25	00:03-00:19	00:06-03:36	00:01-02:41

Wait To Leave ED After Admission	22	17	44	22	0:44	0:45	0:23	0:25	0:24	0:36	0:12	0:08	0:30	1:07	1:07	0:35	00:00-03:08	00:00-02:38	00:00-01:49	00:00-03:33

Wait To Leave ED After Discharge	18	12	23	26	0:02	0:06	0:02	0:07	0:00	0:00	0:00	0:00	0:10	0:01	0:04	0:19	00:00-00:26	00:00-00:46	00:00-00:32	00:00-01:01

Total Time In Area	40	106	82	55	2:47	3:53	3:40	2:57	2:42	3:10	3:27	2:57	2:16	2:05	2:45	3:17	00:10-05:56	00:04-16:45	00:14-11:26	00:14-07:34

Total Time In ED	29	30	64	45	3:07	4:52	4:02	3:22	2:51	4:17	3:49	2:59	2:40	2:42	2:36	3:25	01:05-05:56	00:17-11:58	00:31-11:26	01:15-07:57

C.O., Casualty Officer; CT, Computed Tomography; US, Ultrasound; ED, Emergency Department; SD, Standard Deviation;; IQR, Inter Quartile Range; T_1_, 0200-0800; T_2_, 0800-1400; T_3_, 1400-2000; T_4_, 2000-0200

**Table 2:** Waiting and interaction times in hours and minutes in priority 2

	NUMBER				MEAN				MEDIAN				IQR				RANGE			
	
	T_1_	T_2_	T_3_	T_4_	T_1_	T_2_	T_3_	T_4_	T_1_	T_2_	T_3_	T_4_	T_1_	T_2_	T_3_	T_4_	T_1_	T_2_	T_3_	T_4_
Wait To Enter Triage	38	108	87	113	0:11	0:14	0:13	0:11	0:06	0:10	0:10	0:08	0:13	0:15	0:12	0:11	00:00-00:51	00:00-01:03	00:00-01:14	00:00-01:35

Triage Time Taken	38	108	88	115	0:03	0:04	0:05	0:04	0:03	0:03	0:04	0:04	0:02	0:04	0:04	0:04	00:01-00:12	00:01-00:18	00:01-00:24	00:00-00:19

Wait To Enter Area	37	103	86	112	2:11	1:23	2:24	2:38	1:38	1:18	2:25	2:21	2:36	1:04	2:00	2:08	00:05-07:23	00:02-03:50	00:00-07:00	00:00-22:44

Wait From Registration To First Assessment	37	103	83	105	2:30	1:45	2:45	3:05	1:55	1:41	2:50	2:46	2:44	1:06	2:14	2:14	00:00-07:44	00:14-03:59	00:12-06:07	00:04-03:27

Wait To Be Seen By Nurse 1	6	32	24	18	0:09	0:08	0:08	0:05	0:07	0:04	0:05	0:03	0:10	0:08	0:04	0:04	00:02-00:20	00:00-00:39	00:00-01:10	00:01-00:20

Total Interaction Time Nurse	6	30	23	18	0:11	0:11	0:09	0:08	0:13	0:10	0:06	0:03	0:10	0:13	0:02	0:06	00:04-00:20	00:01-00:47	00:01-01:10	00:01-00:31

Wait To Be Seen By CO 1	38	110	74	92	0:10	0:12	0:11	0:11	0:07	0:10	0:09	0:10	0:07	0:10	0:10	0:11	00:02-00:36	00:01-02:06	00:00-00:58	00:01-00:46

Total Interaction Time CO	38	112	78	94	0:18	0:20	0:21	0:19	0:17	0:16	0:16	0:16	0:17	0:19	0:19	0:19	00:02-00:49	00:01-02:06	00:01-01:18	00:01-01:39

Time First Seen To Time Last Seen By CO	38	112	81	94	1:07	0:56	1:18	2:31	0:35	0:41	0:55	0:35	1:09	1:09	1:27	1:21	00:02-10:40	00:01-03:31	00:01-05:19	00:01-23:48

Wait For Senior Review	1	17	23	25	1:10	1:11	1:13	1:22	1:10	0:57	0:52	1:04	-	0:42	0:39	1:06	1:10-1:10	00:08-03:04	00:01-03:42	00:06-04:52

Total Time X-ray Taken	23	64	48	61	0:14	0:20	0:18	0:14	0:10	0:15	0:14	0:11		0:12	0:11	0:08	00:04-00:45	00:04-01:33	00:02-01:12	00:03-00:55

Total Time CT Taken	-	1	4	6	-	1:15	0:30	0:31	-	1:15	0:25	0:27	-	-	-	0:22	-	01:15-01:15	00:15-00:55	00:09-01:10

Total Time US Taken	-	6	4	1	-	0:56	0:21	0:10	-	0:25	0:13	0:10	-	0:24	0:11	-	-	00:08-02:36	00:08-00:50	00:10-00:10

Total Treatment Time	4	9	12	6	0:08	0:06	0:24	0:02	0:09	0:05	0:08	0:02	0:02	0:07	0:37	0:02	00:04-00:10	00:02-00:14	00:01-01:20	00:01-00:06

Wait To Leave ED After Admission	34	86	67	90	0:58	1:09	1:35	1:29	0:30	0:38	1:06	0:53	1:17	0:59	0:22	0:33	00:00-06:29	00:00-09:05	00:00-07:13	00:00-02:05

Wait To Leave ED After Discharge	18	24	16	38	0:15	0:25	0:15	0:34	0:10	0:11	0:06	0:11	0:04	0:06	0:04	0:11	00:01-01:15	00:01-04:16	00:01-00:52	00:01-02:58

Total Time In Area	34	85	60	83	1:25	1:51	2:29	2:09	1:00	1:28	2:02	1:37	1:12	1:28	2:00	1:26	00:03-07:13	00:05-10:03	00:05-08:07	00:01-02:59

Total Time In ED	32	77	55	79	3:54	3:29	4:38	4:45	3:21	3:02	4:24	4:01	3:05	2:29	2:51	2:47	00:43-08:26	00:56-12:52	00:09-08:28	00:16-04:55

C.O., Casualty Officer; CT, Computed Tomography; US, Ultrasound; ED, Emergency Department; SD, Standard Deviation;; IQR, Inter Quartile Range; T_1_, 0200-0800; T_2_, 0800-1400; T_3_, 1400-2000; T_4_, 2000-0200

**Table 3:** Waiting and interaction times in hours and minutes in priority 3

	NUMBER				MEAN				MEDIAN				IQR				RANGE			
	
	T_1 _	T_2 _	T_3 _	T_4 _	T_1 _	T_2 _	T_3 _	T_4 _	T_1 _	T_2 _	T_3 _	T_4 _	T_1_	T_2_	T_3_	T_4_	T_1 _	T_2 _	T_3 _	T_4 _
Wait To Enter Triage	35	124	103	69	0:10	0:12	0:11	0:11	0:05	0:10	0:10	0:10	0:10	0:10	0:11	0:11	00:01-00:47	00:00-01:07	00:00-01:12	00:00-00:38

Triage Time Taken	35	124	103	69	0:02	0:02	0:01	0:02	0:02	0:02	0:02	0:02	0:01	0:01	0:01	0:01	00:01-00:11	00:00-00:16	00:00-00:09	00:01-00:07

Wait To Enter Area	27	121	100	58	2:38	1:44	2:11	1:58	2:39	1:04	2:17	1:45	2:01	1:17	2:12	1:51	00:33-04:53	00:00-16:58	00:01-04:44	00:05-05:21

Wait To Be Assessed	28	125	102	58	2:43	1:59	2:25	2:14	2:44	1:19	2:40	2:05	-	-	-	-	00:06-05:28	00:02-17:12	00:00-05:04	00:14-05:24

Wait To Be Seen By Nurse 1	11	39	33	23	0:26	0:45	0:35	0:26	0:24	0:39	0:32	0:19	0:12	0:35	0:29	0:24	00:04-00:48	00:00-03:12	00:01-02:20	00:00-01:25

Total Interaction Time Nurse	11	39	33	22	0:11	0:07	0:09	0:08	0:11	0:04	0:04	0:06	0:09	0:06	0:07	0:05	00:02-00:26	00:01-00:44	00:01-01:03	00:01-00:24

Wait To Be Seen By CO 1	34	126	102	63	0:00	0:05	0:02	0:04	0:00	0:00	0:00	0:00	0:00	0:02	0:03	0:03	00:00-00:09	00:00-02:16	00:00-00:45	00:00-00:46

Total Interaction Time CO	34	124	94	60	0:14	0:12	0:11	0:08	0:06	0:08	0:08	0:06	0:08	0:09	0:07	0:08	00:01-02:12	00:01-01:39	00:01-00:50	00:01-00:58

Time First Seen To Time Last Seen By CO	34	128	103	63	0:33	0:27	0:30	0:19	0:22	0:20	0:24	0:11	0:38	0:33	0:32	0:27	00:01-02:35	00:00-03:02	00:00-03:22	00:00-01:33

Wait For Senior Review	3	5	5	3	1:09	0:47	0:34	0:28	0:34	0:33	0:31	0:26	-	0:25	0:28	-	00:29-02:26	00:08-02:24	00:05-01:17	00:13-00:46

Total Time X-ray Taken	29	73	78	46	0:12	0:18	0:18	0:14	0:10	0:16	0:16	0:12	0:07	0:11	0:13	0:10	00:03-00:40	00:02-01:27	00:01-01:21	00:03-00:54

Total Time CT Taken	-	-	-	-	-	-	-	-	-	-	-	-	-	-	-	-	-	-	-	-

Total Time US Taken	-	3	1	-	-	0:14	0:14	-	-	0:15	0:14	-	-	-	-	-	-	00:14-00:15	00:14-00:14	-

Total Treatment Time	9	27	23	14	0:25	0:09	0:15	0:12	0:12	0:05	0:08	0:07	0:10	0:04	0:06	0:04	00:02-02:12	00:02-00:54	00:01-01:44	00:01-00:51

Wait To Leave ED From Admitted	2	7	3	2	0:33	0:48	2:36	0:36	0:33	0:30	0:34	0:36	-	0:26	-	-	00:20-00:46	00:05-02:34	00:03-07:11	00:17-00:56

Wait To Leave ED From Discharge	19	43	38	31	0:24	0:19	0:21	0:22	0:14	0:08	0:14	0:15	0:17	0:09	0:16	0:18	00:00-02:31	00:01-02:18	00:01-01:27	0:01-02:10

Total Time In Area	32	88	81	57	1:55	0:52	1:04	0:40	0:42	0:33	0:38	0:34	0:54	0:32	0:42	0:35	00:05-22:33	00:04-05:25	00:05-22:31	00:00-03:35

Total Time In ED	30	86	80	52	4:08	2:41	3:24	3:06	4:42	2:07	3:44	2:53	4:58	2:11	3:52	2:59	00:58-06:52	00:13-10:29	00:17-08:44	00:28-07:22

C.O., Casualty Officer; CT, Computed Tomography; US, Ultrasound; ED, Emergency Department; SD, Standard Deviation; IQR, Inter Quartile Range; T_1_, 0200-0800; T_2_, 0800-1400; T_3_, 1400-2000; T_4_, 2000-0200

As shown in table 4, characteristics predictive of waiting time were: priority assigned, request for laboratory tests, request for imaging, consultation with a senior registrar (area 1 admitted p = 0.037, area 2 admitted p = 0.0006), and time of arrival (table 5).

**Table 4:** Procedures predictive of prolonged length of stay (LOS)

Causes of Prolonged LOS	Number		Mean LOS (hrs)		T test	P value
	**Yes**	**No**	**Yes**	**No**		

**Presented at ED between 0200 and 1400**	377	349	03:34	03:57	-2.335	0.002

**Laboratory tests ordered in area**	171	569	05:10	03:40	4.980	< 0.001

**Imaging ordered in area**	514	224	04:31	03:51	3.100	0.002

**Seen by senior for admission/discharge**	162	564	04:44	03:28	6.275	< 0.001

**Table 5:** Waiting and interaction times from 0800-0800 in priority 1, 2 and 3

	Area 1					Area 2					Area 3				
	
	Number	Mean	SD	Median	Range	Number	Mean	SD	Median	Range	Number	Mean	SD	Median	Range
Wait To Enter Triage	185	0:08	0:09	0:08	00:00-01:06	346	0:12	0:12	0:09	00:00-01:35	491	0:11	0:10	0:10	00:00-01:12

Triage Time Taken	244	0:04	0:03	0:04	00:00-00:19	349	0:04	0:03	0:03	00:00-00:24	491	0:02	0:01	0:02	00:00-00:16

Wait To Enter Area	185	0:20	0:40	0:03	00:00-04:24	338	2:09	1:36	1:59	00:00-22:44	306	2:00	1:56	1:36	00:00-16:58

Wait From Registration To First Assessment	269	0:52	0:50	0:35	00:00-04:28	328	2:31	1:42	02:18	00:00-03:27	314	2:14		1:46	00:00-17:12

Wait To Be Seen By Nurse 1	205	0:34	0:58	1:56	00:00-04:52	80	0:07	0:09	0:04	00:00-01:10	106	0:36	0:29	0:32	00:00-03:12

Wait To Be Seen By Nurse 2	80	1:10	1:12	0:42	00:02-05:27	21	0:07	0:05	0:07	00:01-00:24	8	0:19	0:19	0:13	00:02-01:03

Wait To Be Seen By Nurse 3	29	1:05	1:54	0:34	00:01-10:02	8	0:03	0:01	0:03	00:00-00:08	1	2:23	-	2:23	02:23-02:23

Total Interaction Time Nurse	185	0:54	1:27	0:54	00:01-13:20	77	0:10	0:12	0:08	00:01-01:10	105	0:08	0:09	0:05	00:01-01:03

Wait To Be Seen By CO 1	352	0:33	0:49	0:13	00:00-00:40	314	0:11	0:10	0:09	00:00-02:06	325	0:03	0:12	0:00	00:00-02:16

Wait To Be Seen By CO 2	220	0:51	1:22	0:25	00:00-03:14	202	0:08	0:08	0:05	00:01-01:05	178	0:25	0:17	0:22	00:01-01:35

Wait To Be Seen By CO 3	110	0:45	0:56	0:24	00:01-04:57	89	0:07	0:06	0:05	00:00-00:31	28	0:26	0:33	0:14	00:02-02:19

Total Interaction Time CO	336	0:23	0:18	0:20	00:01-01:55	322	0:19	0:15	0:16	00:01-02:06	313	0:11	0:13	0:08	00:01-02:12

Time First Seen To Time Last Seen By CO	340	1:21	1:33	0:55	00:01-10:33	325	1:28	2:27	0:38	00:01-23:28	329	0:27	0:30	0:19	00:00-03:22

Wait For Senior Review	99	1:15	1:57	0:46	00:00-04:57	66	1:14	1:02	1:00	00:01-04:52	16	0:43	0:43	0:32	00:05-02:26

Time First Seen To Time Last Seen By Senior	125	0:24	0:34	0:10	00:00-02:53	73	0:20	0:37	0:09	00:01-05:20	24	0:12	0:12	0:07	00:01-00:40

Total Interaction Time Senior	120	0:13	0:09	0:09	00:01-01:35	73	0:09	0:07	0:08	00:01-00:38	24	0:08	0:08	0:04	00:01-00:30

Total Time X-ray Taken	244	0:17	0:13	0:15	00:03-01:28	196	0:16	0:13	0:12	00:02-01:33	226	0:17	0:12	0:13	00:01-01:27

Total Time CT Taken	23	0:17	0:13	0:13	00:05-01:02	11	0:45	0:19	0:27	00:09-01:15	0	-		-	-

Total Time US Taken	21	0:21	0:17	0:14	00:07-01:17	11	0:29	0:40	0:13	00:08-02:36	4	0:14	0:00	0:13	00:14-00:15

Total Time Taken For 2 Imaging Investigations	25	0:22	0:17	0:16	00:04-01:09	2	0:14	0:07	0:14	00:09-00:19	1	0:19	0:19	0:19	00:19-00:19

Time ECG Taken	174	0:03	0:02	0:03	00:00-00:23	84	0:03	0:02	0:03	00:01-00:26	14	0:03	0:01	0:03	00:02-00:08

Total Treatment Time	65	0:45	0:53	0:18	00:01-03:36	31	0:10	0:09	0:06	00:01-01:20	73	0:13	0:20	0:07	00:01-02:12

Wait To Leave ED After Admission	152	0:36	0:47	0:19	00:00-03:33	277	1:18	1:29	0:45	00:00-02:05	14	1:07	1:51	0:32	00:03-07:11

Wait To Leave ED After Discharge	88	0:05	0:15	0:00	00:00-01:50	96	0:22	0:33	0:10	00:01-04:16	131	0:21	0:28	0:12	00:00-02:31

Total Time In Area	283	3:29	2:29	3:08	00:04-16:45	262	1:59	1:58	1:32	00:01-02:59	258	1:01	2:11	0:36	00:00-22:33

Total Time In ED	220	3:48	2:23	3:25	00:04-14:14	243	4:11	2:22	3:41	00:09-04:55	248	3:11	1:45	3:03	00:13-10:29

C.O., Casualty Officer; CT, Computed Tomography; US, Ultrasound; ED, Emergency Department; SD, Standard Deviation

The time for a patient to be assessed for the first time by a casualty officer or nurse differed between areas. Means were: area 1 patients, 52 minutes; area 2 patients, 151 minutes; and area 3 patients, 134 minutes. The total length of stay was also different, with patients in area 1 spending 228 minutes in the ED, patients in area 2 spending 251 minutes and patients in area 3 spending 191 minutes.

**Figure 1:**
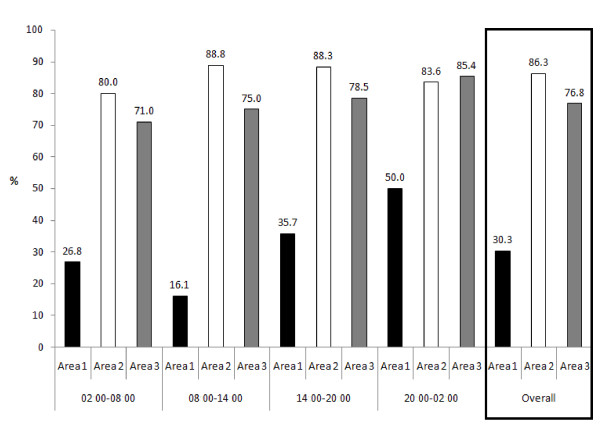
**Patients waiting more than 60 minutes for first assessment**.

**Figure 2:**
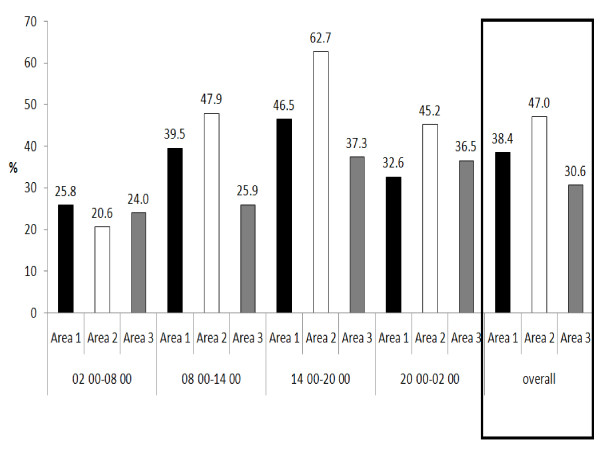
**Patients waiting more than 240 minutes to be admitted/discharged**.

Figure 1 shows the percentage failure rate of reaching the one hour target for first assessment. 30.3% of priority 1 patients, 86.3% of priority 2 patients and 76.8% of priority 3 patients waited more than 1 hour for first assessment. Figure 2 shows that 38.4% of area 1, 47% of area 2 and 30.7% of area 1 patients failed to be admitted/discharged and leave the ED after 4 hours, which is the stated target in the UK guidelines.

## Discussion

There was no delay in waiting time to enter triage and there were no significant differences in median and mean waiting times to enter triage in the different priority patients. This was also true of total time spent in triage. This means that the first contact with the patient is well-managed.

As expected, high priority patients did not wait long to be called into area 1, with a median of 3 minutes waiting time (mean = 20 minutes). In comparison, priority 2 patients spent a median of 1 hour 59 minutes (mean = 2 hours 9 minutes) waiting to be called. Priority 3 patients however, spent a median of 1 hour 36 minutes waiting to enter the area (mean = 2 hours). As expected, priority 2 patients waited longer than priority 1 patients. However, they did not wait less than priority 3 patients in spite of the supposedly greater severity of condition. Similarly, priority 1 patients waited for much less (35 minutes median) than priority 2 or 3 patients (1 hour 42 minutes median, 1 hour 46 minutes median respectively) to be assessed by a casualty officer or nurse for the first time.

Patients were reviewed and re-reviewed by casualty officers and nurses on a regular basis, albeit with occasionally long intervals between such visits. Doctors and nurses dealt with many patients simultaneously, resulting in increased waiting times (tables 1, 2 and 3). As expected, the interaction times (where a casualty officer or nurse is assessing a patient) can be seen to decrease across the three priorities, with mean doctor interaction times varying from 20 minutes (mean 23 minutes) in area 1 to 8 minutes (mean 11 minutes) in area 3. Area 3 was manned by one doctor and one nurse, who saw successive patients as they were called into the area. Thus, no waiting time was required.

Patients requiring admittance when a senior doctor was not present in the emergency department (usually after 2 pm) had to wait for a specialist to come to the department when called. This procedure resulted in delays (table 4), with patients assessed by a senior staying for longer at the emergency department. The wait for specialist review was approximately one hour. Across all areas, imaging (X rays, CTs, and US) took approximately the same time, though it was noted that priority 2 patients wait on average 28 minutes more than priority 1 patients for a CT scan.

As was expected, patients are indeed spending a long time in the ED (tables 1, 2 and 3), and priority 2 and 3 patients spend half that time waiting to be called into their area. Priority 2 patients also spent more time in the ED department in the afternoon than in the morning.

Figures 1 and 2 provide evidence for the need of improvement: graphs showing the percentage of patients who failed to be seen within 1 hour or leave A&E within 4 hours (Figures 1 and 2). However, it would be difficult to attain these targets with the present levels of staff at the MDH ED. 30% of priority 1 and 86% of priority 2 patients waited for more than 1 hour for their first assessment (Figure 1). This might also indicate a failure in the triage system. The latter is not standardized/guided by protocols and very subjective, depending on the experience of nurses conducting triage. The criteria available are not used regularly, and this was noted to result in patients being subjectively moved up or down priorities, as different nurses may have different opinions about how urgent the patient's situation is.

Emergency cases requiring ambulances and resuscitation take up a significant number of staff and this may result in the slowing down of activity in the ED.

It was noted that a very large number of cases attending the ED could have been treated in the primary sector. This applies particularly to limb injuries that need to be X-rayed to exclude or confirm fractures, as evidenced by the large number of patients who presented with traumatic injuries and were referred from Health centres to get an X-ray. X-ray services are not always available in every Health Centre around Malta, since a 24 hour radiographer is not available, and such patients are referred from Health Centres to MDH ED, greatly increasing overall waiting times. A public campaign as to what constitutes an emergency was in place during the study period [11], but this did not seem sufficiently informative, with patients not needing emergency attention continuing to attend MDH ED in large numbers. In fact, although all health centres were closed on the 27th of August, this made no difference to the amount of people who presented at the Emergency Department when compared to the number of attendees on other days.

## Conclusion

Clearly, there is a significant waiting time for patients awaiting review or admission. A significant proportion of patients waited more than 1 hour for first assessment. This exceeds UK benchmarks, and is of detriment to the patient. The system needs to be improved for their benefit. The most obvious improvement that may be implemented is increasing the staff present at the ED department at any one time, especially senior staff who greatly influence patient waiting times. It appears that Mater Dei Hospital does not have enough beds for such a high demand, so that LOS is increased substantially for patients waiting for beds to be available wards. A major contributing factor to experienced delay is the high number of patients presenting that might be easily managed at the primary sector. More investment in primary care (such as 24 hour availability of X-ray machines at health centres) could also be implemented so as to substantially reduce the number of patients. A wider campaign as to what actually constitutes an emergency should be developed so as to inform people when it is appropriate to seek emergency treatment. The triage system may be improved so that patients who do not require hospital can be diverted to health centres. Alternatively, a more stringent and strictly enforced criterion for admission could significantly improve waiting times for patients who truly need it. A new system could also involve the reviewing of patients in the waiting area, as patient condition may improve or deteriorate during the waiting period.

### Limitations

Sources of error in the study included:

• Staff might have unconsciously performed differently during the study week due to our presence in the ED (Hawthorne effect).

• Some patients were treated in the decontamination rooms, on stretchers in the stretcher bay or corridors. These were difficult to monitor and might have had some data missing, necessitating their being left out of the study.

• Many patients bypassed triage or registration and were entered straight to the examination areas, and could not be included in the study.

• The study was carried out during the summer months, which have different admission patterns compared to the rest of the year. Future studies would ideally be conducted throughout the year.

## Abbreviations

A&E: accident and emergency; ED: emergency department; LOS: length of stay; MDH: Mater Dei Hospital.

## Competing interests

The authors declare that they have no competing interests.

## Authors' contributions

MC, MA, KC, RE, all participated in data gathering and were involved in the planning of the audit. All were involved in the inputting of data into a database and the interpretation of the data. Students acknowledged below were involved in data gathering. MC drafted the manuscript. VG carried out the statistical interpretation involved and was involved in the planning of the audit. CMA was also involved in the planning of the audit. All authors read and approved the final manuscript.
